# Enterocutaneous fistula due to mesh fixation in the repair of lateral incisional hernia: a case report

**DOI:** 10.1186/1757-1626-1-370

**Published:** 2008-12-02

**Authors:** Sarath Chandra Sistla, Rajesh Reddy, Kadambari Dharanipragada, Sadasivan Jagdish

**Affiliations:** 1 Department of Surgery, Jawaharlal Institute of Postgraduate Medical Education and Research, Pondicherry, India

## Abstract

Enterocutaneous fistula following mesh repair of incisional hernia is usually due to mesh erosion of the underlying viscus and presents late. We describe an early enterocutaneous fistula due to an unusual but a potential mode of bowel injury during mesh fixation. This case is reported to emphasize the need for greater attention to the technique of mesh fixation. We suggest laparoscopic guidance to prevent this serious complication in lateral Incisional hernias with ill defined edges of the defect.

## Background

Enterocutaneous fistula is a late complication of mesh repair of incisional hernia and is usually due to erosion of intestines by the mesh. Early development of enterocutaneous fistula is usually due to inadvertent and unrecognized enterotomy during separation of adhesions. We present our experience with an enterocutaneous fistula due to anchoring stitch resulting in bowel injury during mesh fixation in an onlay repair of a lateral Incisional hernia. Ill defined edges of the hernial defect and bowel adherent to the sac were the cause of this complication. A simple technical solution to avoid this serious complication is suggested.

## Case history

**Figure 1:**
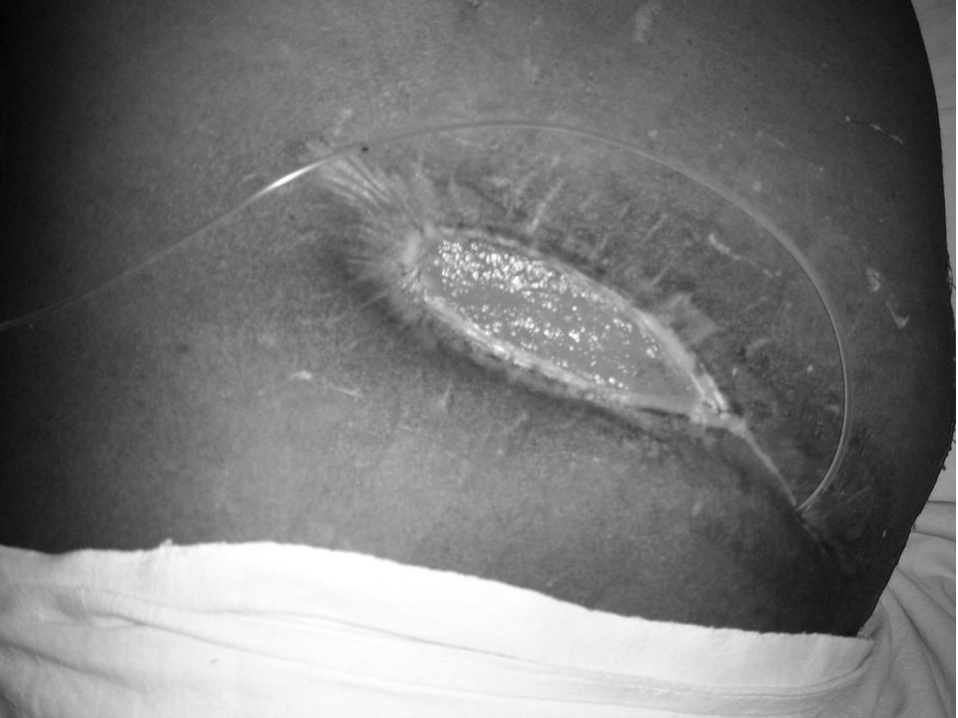
Enterocutaneous fistula at the medial end of the wound.

**Figure 2:**
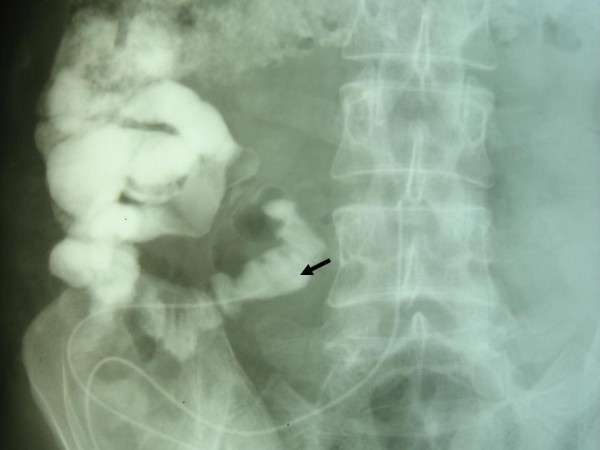
Fistulogram demonstrating communication with the bowel.

A 50 year old man developed incisional hernia following right lumbar sympathectomy for peripheral vascular disease. The incisional hernia was repaired by onlay meshplasty using a polypropylene mesh (6" × 6") and the mesh was anchored to the aponeurosis beyond the defect with polypropylene sutures. The hernial sac was not opened as he did not have any symptoms of intestinal obstruction. On the 5^th ^postoperative day, patient developed deep surgical site infection with *E. coli *necessitating removal of mesh on 14^th ^postoperative day. No difficulty was encountered in the removal of the mesh as it was done in the early postoperative period. Subsequently the patient developed a persistent discharging sinus at the medial end of the wound (Figure 1). Fistulogram revealed communication with ileum (Figure 2). On exploration by a lower right paramedian incision, a loop of small bowel was found to be adherent to the sac with a small fistulous opening on the antimesenteric border. The anchoring suture was found to have gone through the bowel at the site of the enterocutaneous fistula. The bowel loop was detached from parietes and repaired following which the patient made an uneventful recovery.

## Discussion

Repair of incisional hernia is done by either suture repair or prosthetic mesh repair. The latter is associated with a lower recurrence rate but a higher incidence of complications. The most serious complication though fortunately rare, is the development of enterocutaneous fistula [1,2].

Enterocutaneous fistula is a late complication of prosthetic mesh repair of incisional hernia and is usually due to chronic erosion of bowel by mesh placed in direct contact with intestinal loops both in open and laparoscopic repairs [3-5].

Unrecognized bowel injury during release of adhesions and thermal or trocar injuries to bowel in laparoscopic repair can also result in fistula. Basoglu emphasized the need for peritoneal or omentum coverage for prevention of bowel contact with mesh [6].

The incidence of enterocutaneous fistula due to prosthetic mesh is higher in subfascial (5.2%) than in onlay (2.6%) position [7]. In the present case, the patient did not have any symptoms of intestinal obstruction the hernial sac was not opened and it was preserved. Preserving the hernia sac provides a layer of viable autogenous tissue to serve as a barrier between the prosthesis and the intraperitoneal contents, possibly decreasing the risk of adhesions, intestinal obstruction and fistula [8].

The technique described by Khaira et al provides an additional barrier of rectus sheath or aponeurosis between the mesh and the hernial sac and also reduces the risk of bowel injury during mesh fixation [9]. This risk is greater in lateral (transverse or oblique) incisional herniae where the edges of the defect are not as well defined as in midline herniae.

In our case the anchoring suture had injured the bowel which was adherent to the sac. The resultant fistula was well localized with no contamination of the peritoneal cavity. The discharge was not feculent as it involved a very small part of the circumference of the bowel. Enterocutaneous fistula in this case was diagnosed by contrast study. A similar case of enterocutaneous fistula was reported by Acar et al [7].

This case is reported to illustrate the potential risk of bowel injury during mesh fixation in onlay mesh repair, especially in lateral incisional herniae with ill defined edges of the hernial defect. The hernial sac need not be opened routinely in the absence of symptoms of obstruction. Separation of asymptomatic adhesions only increases the risk of bowel injury. We suggest anchoring the mesh under laparoscopic guidance with a 5 mm telescope away from the site of repair. The edges of the defect can be better defined by laparoscopy thereby helping in placing the mesh beyond the defect. Laparoscopic guidance can also prevent accidental injury to bowel during mesh fixation. This also obviates the need for an expensive dual mesh used in laparoscopic repairs.

## Consent

Informed consent of the patient was obtained for publication of this case report. The pictures in this manuscript do not reveal the identity of the patient

## Competing interests

The authors declare that they have no competing interests.

## Authors' contributions

SCS conceived and designed the study. SCS and RR collected the data. SCS drafted the manuscript with the help of KD and JS. SCS, RR, KD and JS were involved in interpretation and analysis of data. All the authors read and approved the manuscript.
